# Capsule Dosimeters for Ultraviolet Radiation Measurements on Coral Reefs and in Seawater

**DOI:** 10.3390/ma16175734

**Published:** 2023-08-22

**Authors:** Elżbieta Sąsiadek-Andrzejczak, Malwina Jaszczak, Marek Kozicki

**Affiliations:** Department of Mechanical Engineering, Informatics and Chemistry of Polymer Materials, Faculty of Material Technologies and Textile Design, Lodz University of Technology, Żeromskiego 116, 90-543 Lodz, Poland; malwina.jaszczak@p.lodz.pl (M.J.); marek.kozicki@p.lodz.pl (M.K.)

**Keywords:** coral reefs, seawater, UV radiation, radiochromic dosimeter, UV dosimeter, TTC-Pluronic F-127 dosimeter

## Abstract

This work reports on the new chemical dosimeters for UV radiation dose measurements on coral reefs and in seawater. The proposed dosimeters can measure the actual dose of UV radiation, which consists of 95% UVA and 5% UVB radiation, unlike the currently-used radiometers in marine and ocean waters that measure the dose of UVA and UVB radiation separately. The dosimeters are composed of water, poly(ethylene oxide)-*block*-poly(propylene oxide)-*block*-poly(ethylene oxide) (Pluronic F-127) as a gel matrix, and 2,3,5-triphenyltetrazolium chloride (TTC) as a UV radiation-sensitive compound. In the work, the dosimeters were characterised in terms of their response to the dose of UV radiation depending on the TTC concentration and the irradiation and storage conditions of the dosimeters. The stability of the dosimeters over time was also examined. The obtained results indicate that the TTC-Pluronic F-127 dosimeters can be used to measure absorbed doses of UV radiation in the saltwater environment. The developed dosimeters with a concentration of 0.1% TTC can be used up to 5 J/cm^2^, which predisposes them to UV radiation measurements at a depth of more than 10 m in sea and ocean waters in 10-min intervals during all months throughout the year.

## 1. Introduction

Coral reefs are most common in shallow, sunny waters of warm seas and oceans on both sides of the equator, mainly along the western coasts of the Atlantic and Indian Oceans and around islands located in the Pacific Ocean. The coral reef is one of the most biodiverse ecosystems in the world. Despite occupying less than 1% of the surface of the seas and oceans, they are the living environment for approximately 25% of marine animals [1].

Unfortunately, the coral reef is a very sensitive ecosystem to environmental changes resulting from natural causes, including UV radiation [2,3,4,5] and human activity. Recently, more attention has been paid to the bleaching of reefs, which results from global warming and is directly related to the increase in the average seawater temperature. One of the main impacts of exposure to UV radiation on the marine ecosystem is changes in genetic material, reproductive performance, metabolism, and pigmentation for a wide range of living organisms such as phytoplankton, seagrasses, algae, corals, and fish [6,7,8]. However, the range of effects of UV radiation depends on the time of day and exposure time (among other factors), which result from latitude, season, and weather conditions [9,10]. In addition, atmospheric pollutants, such as mineral dust, sea salt, or sulfuric acid, can reduce the amount of UV radiation, especially UVB, that reaches the surface of seawater and oceans. UV radiation penetration in the marine environment depends on the optical properties of the seawater and the amount and characteristics of dissolved organic matter, phytoplankton, and other suspended particles. Terrific inputs, upwelling events, and variations in dissolved organic matter can modify the optical properties of seawater due to absorption and scattering, which affect the attenuation of both photosynthetically active radiation (PAR; 400–700 nm) and UV radiation. Additionally, a factor that is not well researched and described is the phenomenon of water lensing, which can be visualised on coral reefs as ripples of high irradiance and its UVB, UVA, and PAR components [3,9]. Water lensing, the optical interaction of light with fluids and aquatic surfaces, is a complex phenomenon. The effect is readily observable above 71% of Earth’s surface in aquatic systems, particularly in shallow marine reef environments. As visible light interacts with aquatic surface waves, time-dependent nonlinear optical aberrations appear, forming caustic bands of light on the seafloor and producing refractive lensing that magnifies and demagnifies underwater objects.

UV measurements on coral reefs are made using radiometers in conjunction with optical measurements and numerical radiation transfer models to better determine changes in UVA and UVB penetration with depth. Radiometers measure the absorbed dose of UVA or UVB radiation, while UV radiation reaching the Earth contains both about 95% UVA and 5% UVB. An alternative to such UV measuring devices may be recording the cumulative dose of UVA and UVB radiation to reflect the actual irradiation conditions. UVA or UVB radiometers monitor UV radiation in the long term and, for this reason, they are connected to a global network using satellite detection techniques [10,11]. These calculations do not require any actual field work, saving a lot of time, effort, and money. However, the precision of UV exposure calculations limits the applicability of UV models, particularly during unforeseen events like storms, hurricanes, extreme tidal events, and ocean pollution, e.g., oil spills from sea transport. The accuracy of numerical models is related to the availability and quality of input data, e.g., the optical properties of seawater and its components (which can change to a large extent in a short period of time). In addition, in underwater conditions, the use of radiometers and spectrometers becomes difficult, with routine calibration techniques, the immersion effect, power requirements, and maintenance becoming more problematic than when working on land. Thus, in order to properly estimate the underwater radiation intensity and exposure to UV radiation, these techniques should be supplemented with other measurement techniques [11] because actinometers are just instruments for measuring radiation intensity and are used in meteorology to measure solar radiation as pyranometers, pyrheliometers, and net radiometers. For this purpose, dosimeters in the form of devices or systems able to register absorbed radiation doses that can be compared with a standard are used. Biological and chemical dosimeters are examples of such a solution, but their use is more difficult to calibrate and measure with conventional equipment [12]. Thus, they are not suitable for long-term measurements. In addition, their preparation for use and analysis requires significantly more time and complexity compared to their chemical counterparts. The most commonly used chemical UV actinometers are based on the photolysis of nitrites and nitrates. To measure UV exposure underwater at different depths, a scalar photon exposure with a very high level of sensitivity is used, with an accuracy similar to that of spectroradiometric measurements. However, the preparation, use, and reading of such a dosimeter require the work of qualified people and the use of specialised equipment for degradation analysis [13]. Studies with poly(2,6-dimethyl-1,4-phenylene oxide) (PPO) films have shown that they can be used in the air to measure large amounts of UV exposure over extended periods of time. Based on the optical properties of these films, including the cosine response, the temperature effect on optical absorbance, spectral responsivity, and exposure reproducibility, these films were found to be suitable for UV measurements for up to about four days under unshaded summer sun in subtropical [14,15]. Another example are dosimeters based on o-nitrobenzaldehyde and polysulfone, which have the ability to record cumulative exposure to UV radiation only at short intervals, usually not longer than two days. Such a short period of time spent in the underwater field environment significantly reduces the amount of data available for analysis. Being able to record high levels of exposure is very important because the biological damage caused by UV radiation to the marine ecosystem is a long-term, cumulative process. Therefore, it was assumed that there is a need for chemical dosimeters that can be easily used in marine applications and that are capable of measuring UV radiation doses over time.

The concept of this work is based on the use of radiochromic hydrogel dosimeters with 2,3,5-triphenyltetrazolium chloride (TTC) as a UV radiation sensitive compound and a Pluronic F-127 matrix for measuring UV radiation in the marine ecosystem. Dosimeters would be placed in the plastic capsules and fixed in the seas and oceans at various depths. Pluronic F-127 was chosen for the dosimeter matrix because it ensures high transparency of the dosimeter and is also non-toxic. Despite placing the dosimeters in capsules, it is necessary to take into account possible damage to them, which can cause the dosimeter to flow out into the seawater. Due to the non-toxicity of Pluronic F-127 and trace amounts of the radiochromic compound, the dosimeter would not be a threat to the aquatic environment. So far, radiochromic hydrogels with Pluronic F-127 have been studied as 1D, 2D, and 3D dosimeters for UV and ionising radiation dose measurements, such as (i) textile dosimeters printed with NBT-Pluronic F-127 pastes [16]; (ii) LCV-Pluronic F-127; (iii) TTC-Pluronic F-127; (iv) LMG-Pluronic F-127 [17]; and (v) NBT-Pluronic F-127 capsules [18].

The aim of this work is to present new capsule dosimeters made of Pluronic F-127 and TTC that can measure the dose of UV radiation at various depths in seawater. The innovation is the fact that, unlike currently used dosimeters that measure the absorbed dose of UVA or UVB radiation, TTC-Pluronic F-127 dosimeters can measure the actual dose of UV radiation, which is the total dose of UVA (95%) and UVB (5%) radiation. At first, TTC-Pluronic F-127 dosimeters containing two different concentrations of TTC were prepared, and the samples were then irradiated with UV radiation and investigated with respect to the obtained calibration and basic characteristics of the dose response. Colour changes due to irradiation were examined for different UV subranges and in air and seawater conditions. Reflectance spectrophotometry was used to analyse the colour of the developed dosimeters. Finally, the possibility of using the TTC-Pluronic F-127 dosimeters on coral reefs and in seawater for measuring the absorbed dose of UV radiation is presented.

## 2. Materials and Methods

### 2.1. Preparation of Samples

Dosimeter samples were composed of distilled water, poly(ethylene oxide)-*block*-poly(propylene oxide)-*block*-poly(ethylene oxide) (Pluronic F-127, Sigma-Aldrich, Saint Louis, MO, USA) as a gel matrix, and 2,3,5-triphenyltetrazolium chloride (TTC, M = 334.81 g/mol; Sigma-Aldrich, Saint Louis, MO, USA) as a radiation-sensitive compound. TTC was chosen as a representative of tetrazolium salts, which showed a response to UV irradiation as reported elsewhere [17]. Pluronic F-127 is approved by the US Food and Drug Administration (FDA) as a nontoxic copolymer [17], which provides a colourless and transparent hydrogel matrix that does not change colour after UV irradiation [17,18]. Depending on ambient temperature and concentration, Pluronic F-127 can form an aqueous solution or a physical gel. For instance, a 25% Pluronic F-127 solution is in the form of a physical gel in the temperature range of 19–85 °C, and at 5 °C, it is a solution that is easily mixed with an aqueous solution of a UV-sensitive compound.

The TTC was dissolved in distilled water at room temperature using a magnetic stirrer. Then, the solution was placed in a refrigerator (5 °C; 15 min) and then mixed with the cooled Pluronic F-127 aqueous solution (33% *w*/*w*) prepared 72 h earlier. The final concentration of Pluronic F-127 in the hydrogel was equal to 25% *w*/*w* and the concentration of TTC was 0.5 and 1 g/dm^3^. The concentration of TTC was established based on our previous work on the use of this compound in UV dosimetry and due to its good stability over time. The procedure for preparing the Pluronic F-127 pre-solution is described elsewhere [17]. Afterwards, the solution was poured into round, polystyrene plastic containers with caps (PS, Roth, Karlsruhe, Germany). The outside diameter of the container was 37 mm, the inside diameter was 33 mm, the thickness of the container (height) was 7 mm, the height of the gel inside the container was ~3 mm, and the volume of gel solution was equal to about 2.5 mL. All TTC-Pluronic F-127 samples were prepared in the same manner. No other methods of sample preparation were used, i.e., gels of different thicknesses, multilayer gels, etc. Thus, the influences of such structures on the response of the dosimeter to the UV dose were not investigated. The shape and dimensions of the containers allowed the manufacture of circular, flat dosimeters that could be easily measured with a reflectance spectrophotometer. In addition, the PS containers reduced the drying of the gel. Then, the samples were covered with aluminium foil to avoid accidental exposure to daylight and stored at room temperature (23 °C). After 15 min, the sol-gel transition occurred. The samples were irradiated with UV radiation sources and measured with a reflectance spectrophotometer.

### 2.2. Preparation of Seawater

To recreate the natural environment for the reef, seawater was prepared from synthetic salt, Aquaforest Reef Salt (Aylesbury, Buckinghamshire, UK), which is recommended for coral breeding, including soft corals, small polyped stony (SPS) corals, large polyp stony (LPS) corals, and clams. The composition of the salt has been selected to create the best conditions for marine animals. The micro- and macro-elements contained in the salt fully satisfy the demand of corals for the elements needed for their proper growth and coloration. The salt was dissolved in the demineralized water. The water temperature was 24 °C. To obtain a salinity of 33 ppt (parts per thousand), 330 g were dissolved in 10 dm^3^ of water. The solution was intensively stirred with a magnetic stirrer (300 rpm; 15 min). The solution was then allowed to stand for 24 h for complete dissolution of the salts. After obtaining full clarity, the salty water was ready for use. According to the Aquaforest Reef Salt data sheet, the composition of water after salt dissolution was as follows: Cl—19,000–19,500 mg/dm^3^; Na—9720–11,880 mg/dm^3^; Mg—1300–1360 mg/dm^3^; S—810–990 mg/dm^3^; Ca—410–430 mg/dm^3^; K—360–380 mg/dm^3^; B—4.05–5.0 mg/dm^3^; Sr—7.2–8.8 mg/dm^3^; °dKH—7.4–8.2; pH—8.0–8.2.

### 2.3. Irradiation of Samples

All TTC-Pluronic F-127 samples (gels in PS containers) were irradiated in UV-curing cabinets (UVP, Upland, CA, USA) at two wavelengths corresponding to UVA (8 W, type F8T5 Blacklight, range 315–400 nm; a peak at 369 nm, Hitachi, Tokyo, Japan), and UVB (8 W, type G8T5E, range: 280–360 nm; a peak at 306 nm, Sankyo Denki, Tokyo, Japan). UV radiation reaching the Earth’s surface comprises approximately 95% UVA and 5% UVB. Therefore, to reflect real conditions, all samples were irradiated with selected UV radiation doses in the range of 0–5 J/cm^2^ in such a way that 95% of the total absorbed radiation dose was UVA and 5% of the total absorbed radiation was UVB. For example, a sample irradiated with a total dose of 1 J/cm^2^ was irradiated with 0.95 J/cm^2^ UVA and 0.05 J/cm^2^ UVB. A given UV dose (J/cm^2^) was delivered automatically using a built-in detector and the control system of the device. The samples were also irradiated in seawater prepared as described in Section 2.2. For this purpose, the containers with the dosimeter in PS were immersed in a container with water to a depth of 15 cm.

To assess the dose-response effect of the TTC-Pluronic F-127 dosimeter and to determine whether polystyrene (PS) is absorbing UV radiation, some samples were also irradiated without plastic caps. Based on the obtained results, it was found that PS caps absorb only a slight amount of UVB and UVA radiation. Thus, the actual dose delivered to TTC-Pluronic F-127 dosimeter samples covered with PS caps is lower by 10 and 12% for UVA and UVB, respectively (the dose (J/cm^2^). This is indicated below in the figures as the dose emitted by the UV irradiators.

### 2.4. Reflectance Measurements

The reflectance spectra of the TTC-Pluronic F-127 samples were measured using a light reflectance instrument (Spectraflash 300, D65/10; 10 nm resolution, the measurement error is 0.1%, DataColor, Rotkreuz, Switzerland). UV light was automatically cut off by the software microMATCH v. 3.6 (UV filter FL40) and manually (0% of UV on the physical UV calibrator) so as not to irradiate the samples during the measurements. The TTC-Pluronic F-127 samples were measured immediately after irradiation and over time after irradiation, over a wavelength range of 400–700 nm. The stability of dosimeters has been studied over a longer period (30 days).

After approximately 20 days, air bubbles appeared in the TTC-Pluronic F-127 gel structure (drying of the hydrogel) for samples that had not been stored in water. However, they did not significantly affect the reflectance measurements and measurement uncertainty of the samples. For example, the difference in the remission of the TTC-Pluronic F-127 sample irradiated with a dose of 0.5 J/cm^2^ without air bubbles and with air bubbles was less than 1%. Afterwards, the wavelength for which the change in reflectance was maximal was selected and discussed vs. the absorbed UV dose. Based on reflectance measurements, the characteristic parameters of TTC-Pluronic F-127 dosimeters were determined: dose sensitivity, linear and dynamic dose-response, and threshold dose. Additionally, the colour coordinates were determined using the CIE Lab evaluation system, which describes the perceived colour according to the ISO/CIE 11664-4 standard [19]. Chromatic colours are described by using the L*a*b* description, where L* represents lightness from black to white on a scale of zero to 100, while the value “a*” represents the green-red axis and the value “b*” represents the blue-yellow axis. 

### 2.5. Stability of Samples

The stability of the TTC-Pluronic F-127 samples was assessed over time before and after UV exposition. The measurements were made for the samples at a temperature of 23 °C. During the experiment, the samples were stored in air and seawater and covered with aluminium foil to limit the drying of the hydrogel and the access of light to the samples. The in-time stability measurements were made for non-irradiated and UV irradiated samples after 1–28 days after preparation or irradiation.

## 3. Results and Discussion

### 3.1. Dose-Response of Hydrogels

The TTC-Pluronic F-127 gel samples containing different concentrations of TTC (0.1 and 0.05% (*w*/*w*)) were irradiated with UV radiation in air and seawater, resulting in a colour change of the gels from colourless to red, caused by the conversion of TTC to red-coloured formazan, according to the reaction shown below (1) [20].

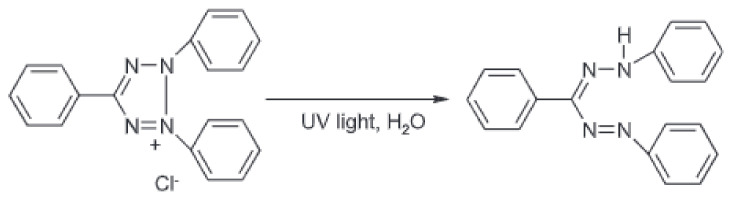
(1)

The intensity of the colour change increases with the absorbed dose of UV radiation. This effect is visible in the photographs in Table 1, showing samples after irradiation with selected doses of UV radiation in the range of 0–2 J/cm^2^.

**Figure 1:**
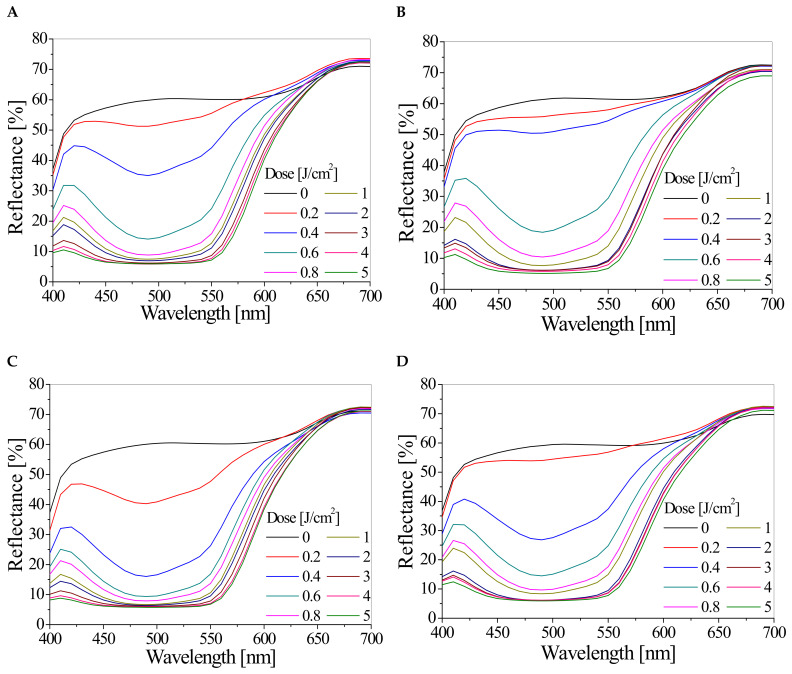
Reflectance spectra of TTC-Pluronic F-127 dosimeters, containing 0.1% (**A**,**C**) and 0.05% (**B**,**D**) TTC, irradiated with UV radiation in air (**A**,**B**) and seawater (**C**,**D**) medium.

Increasingly intense colour changes of the TTC-Pluronic F-127 dosimeters, along with the increase in the absorbed radiation dose, were recorded using the reflectance spectra, which are shown in Figure 1. A decrease in the reflectance of light is observed in the wavelength range of 410–550 nm, with a minimum present at 490 nm. The lower the reflectance values, the greater the absorbed dose of radiation. It was registered (Figure 1, Table 1) that the use of a higher concentration of the radiochromic dye TTC in the gel composition results in a more intense response to the radiation dose. Doubling the concentration from 0.05% to 0.1% resulted in a decrease in the reflectance value of the samples in the tested dose range, and this relation is visible for samples irradiated with UV radiation doses up to 1 J/cm^2^. For example, the reflectance at 490 nm of the sample irradiated in seawater with 0.6 J/cm^2^ of UV radiation is 35% lower for the dosimeter with 0.1% TTC than for the dosimeter with 0.05% TTC. TTC-Pluronic F-127 dosimeters show a different response to the absorbed dose depending on the irradiation environment (air or seawater). Lower reflectance values are recorded for samples irradiated in seawater than in the air (Figure 1, Table 1). For instance, the reflectance at 490 nm of the sample containing 0.1% TTC and irradiated with 0.6 J/cm^2^ of UV radiation in seawater is 33% lower than for the sample irradiated in the air.

Additionally, a comparison of the colour change of samples with different TTC concentrations irradiated with UV radiation in water or air is expressed in the CIELab system and presented in Table 2. The increase in the intensity of the red colour with the increase in the absorbed dose of UV radiation is evidenced by the decreasing L-coordinate value.

**Figure 2:**
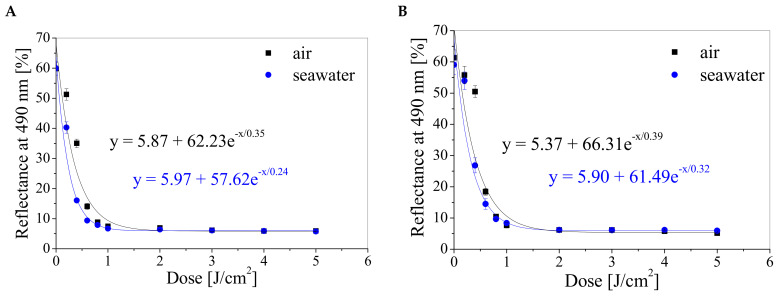

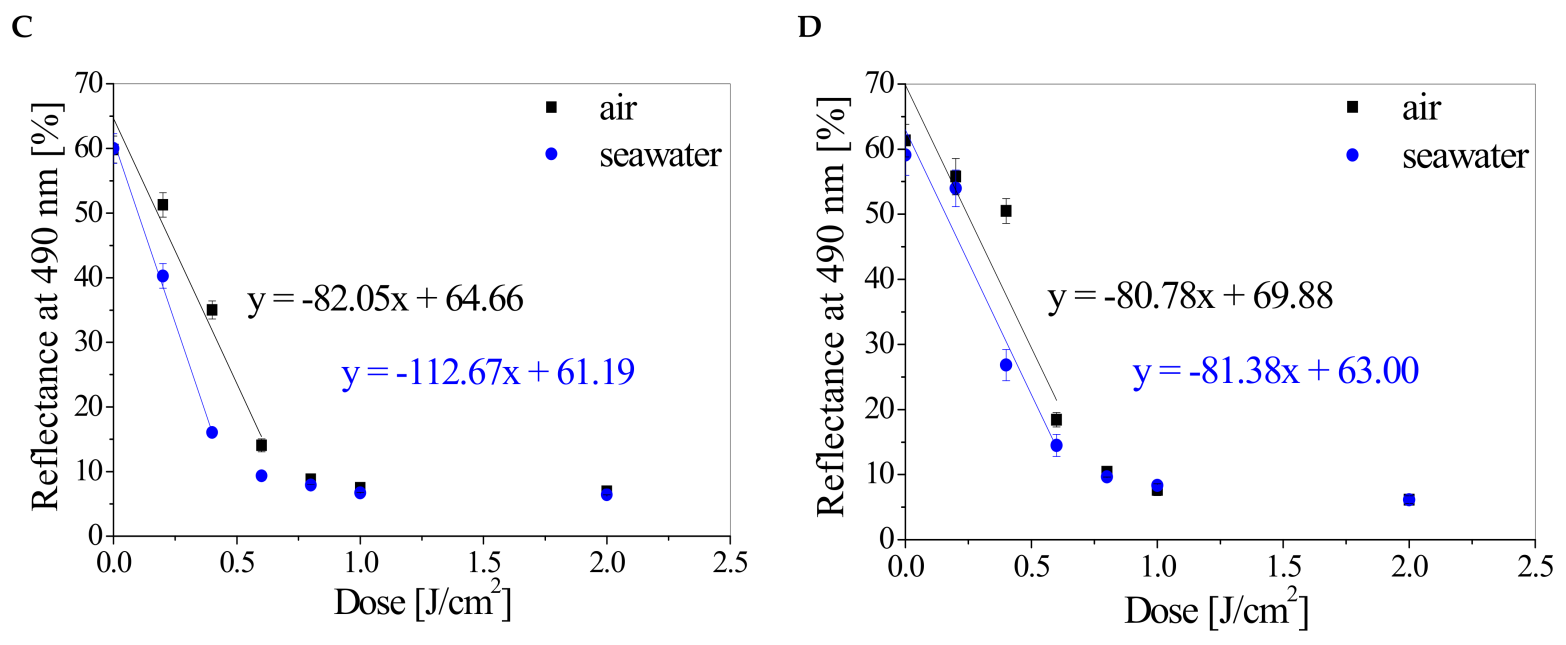
The dose-response of TTC-Pluronic F-127 compositions containing 0.1% TTC (**A**,**C**) and 0.05% TTC (**B**,**D**), irradiated with UV radiation in air and seawater at a specific wavelength, with fitted curves for the dynamic dose ranges (**A**,**B**) and with the determined linear dose ranges (**C**,**D**). Each point on the graph is the average of five measurements.

On the basis of the reflectance spectra shown in Figure 1, the dose-responses of dosimeters expressed as reflectance at 490 nm versus absorbed dose were plotted and presented in Figure 2. The dose-responses are described by exponential equations. Based on the results presented in Figure 2, the main characteristics of the TTC-Pluronic F-127 dosimeters with different TTC concentrations and irradiated with UV radiation in air and seawater, such as dose sensitivity, intercept, threshold dose, linear dose range, and dynamic dose range, were extracted and presented in Table 3. The dosimeters that have been irradiated in seawater and contain a higher concentration of TTC are characterised by a higher sensitivity. For example: (i) the sensitivity of a dosimeter containing 0.1% TTC irradiated with UV in water is 37% higher than when irradiated in air, (ii) the sensitivity of a dosimeter containing 0.1% TTC and irradiated in water is 38% higher than the sensitivity of a dosimeter irradiated in the same way but containing 0.05% TTC. The threshold dose for all gels studied is less than 0.02 J/cm^2^, which indicates that dosimeters react to very low doses of UV radiation. Dosimeters show a wide dynamic dose range from less than 0.2 J/cm^2^ to at least 2 J/cm^2^ and a narrow linear dose range from 0 J/cm^2^ to 0.4 J/cm^2^ or 0.6 J/cm^2^, depending on the TTC concentration and irradiation conditions (Table 3).

The study also analysed the TTC concentration of 0.005% in order to extend the measurement range in the area of the linear and dynamic response of dosimeters to the dose of UV radiation. The use of such a low concentration of TTC in the sample allowed for a significant reduction in the colour intensity after UV irradiation; however, above the dose of 0.5 J/cm^2^, the samples reached the maximum colour saturation. Thus, it became impossible to use the developed dosimeters to measure doses above 1 J/cm^2^. Another way, which is not discussed in this work, to increase the measuring range is to change the pH of the samples, use UV retarders, or use other dye types, e.g., direct dyes.

### 3.2. Stability of Samples

The stability over time of the TTC-Pluronic F-127 gel dosimeters before and after UV irradiation was examined. The reflectance measurements over time were made within 28 days of sample preparation and irradiation, respectively. During this time, the samples were stored at room temperature (23 °C) in a dark, dry place, and for comparison, in seawater. In the structure of the dosimeters stored in the air, the appearance of air bubbles, formed as a result of drying the gel (probably the containers were not perfectly matched to prevent the gels from evaporating), was observed already after 14 days from the production and irradiation (Table 4). This phenomenon has not been observed for samples stored in seawater, which is desirable for the application of these systems as dosimeters for use in seas and oceans.

**Figure 3:**
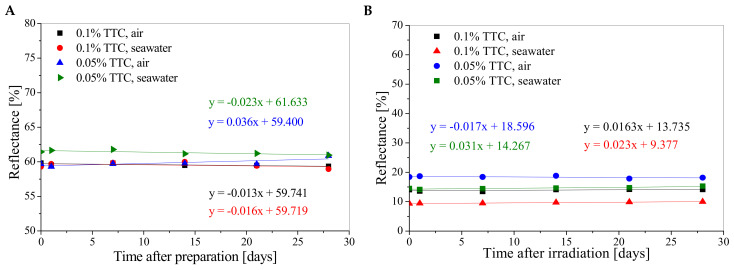
Stability over time of TTC-Pluronic F-127 dosimeters containing 0.1% and 0.05% TTC, unirradiated (**A**) and irradiated with 0.6 J/cm^2^ UV radiation (**B**) and stored in air and seawater conditions.

No change in colour of non-irradiated samples was observed up to 28 days after preparation (Table 4). Changes in reflectance values after 28 days varied by less than 2% for all tested samples (Figure 3A). This is a desirable property of the dosimeter because the spontaneous change of the dosimeter’s colour over time would affect the reliability of reading the absorbed dose of UV radiation. In the case of samples irradiated with UV radiation, slight changes in the colour of the samples are observed over time. The reflectance changes after 28 days for samples with TTC concentrations of 0.1% and 0.05% were, respectively, 0.5% and 1.4% when stored in air and 6.7% and 5.6% when stored in water (Figure 3B). Small changes in colour over time in the irradiated and non-irradiated samples are also confirmed by the slope coefficients of the determined linear equations, which are close to the value of 0 (Figure 3).

### 3.3. Proposition of Application

The proposed dosimeters can be used to measure UV radiation doses in salty waters near coral reefs. The developed dosimeters are used to measure the actual dose of UV radiation cumulatively without dividing the results into UVA and UVB, contrary to the use of radiometers. Thus, the proposed dosimeters will allow for supplementary or independent information to be obtained apart from the commonly-used methods of measuring UV radiation on reefs. Depending on their geographical location, season, and depth, coral reefs absorb different doses of UV radiation. Thus, the concentration of TTC should be adjusted. The greater the insolation in a given region and the shallower the water depth, the lower the recommended TTC concentration. The highest doses are absorbed in the spring and summer months, e.g., for Red Sea reefs, the highest doses are recorded from April to August. However, with increasing depth, lower absorbed UV doses are noted. For example, in the Red Sea in July 2018, at a depth of 10 m, the UVA and UVB daily doses were 59% and 90.7% lower than at a depth of 1 m, respectively [2]. At this point, it is worth noting that the measurements of UV radiation are affected, by the length of exposure and the optical properties of water, among other factors, and thus the optimal time for recording radiation by the dosimeter should be considered. Daily measurements of the UV radiation dose are mainly influenced by the season and the weather, especially the degree of cloudiness. Thus, these measurements are burdened with a high degree of measurement uncertainty, which is difficult to clearly define. In addition, the accuracy of measurements in water is affected by the so-called lens effect, which is also not included in the results. Reducing the measurement time of the dosimeter also minimises problems with chemical dosimeters, such as low stability over time and the possibility of undesirable substances, such as microplastics, getting into the water. The shorter time also minimises the possibility of unsealing the capsules and the risk for living organisms such as fish and squid swallowing the dosimeter. Table 5 shows the UV radiation doses measured in April, June, September, and December at the Red Sea for three depths of 1, 10, and 20 m recorded in 24 h, 1 h, and 10 min [2]. The table summarises data for months selected from each season, taking into account the months with the maximum (July) and minimum (December) exposure to UV radiation.

To assess the possibility of using the developed dosimeters in real conditions, the dosimeter with a concentration of 0.1% TTC was selected due to its wider measurement range up to 5 J/cm^2^. It can be concluded that the dosimeter can be used at a depth of 10 m or below throughout all the months of the year, taking 10-min measurements. Hourly measurements are also possible at depths of 20 m and below during the months of medium and low insolation (from autumn to spring). Depending on the geographical region, season, weather conditions, and the depth of coral reefs, the concentration of TTC should be adjusted. Moreover, in order to extend the scope of application of the dosimeter to shallower depths during all months, the chemical composition of the dosimeters should be modified by the selection of UV radiation retarders. This will be the subject of future studies.

The developed capsule dosimeters (hydrogel in a polystyrene capsule) can be used in the following ways: (i) single capsules attached to ropes at buoys at various depths; (ii) a group of capsules formed as flat packages, attached to the ropes at the buoys.

The use of polystyrene capsules protects the dosimeter against leakage due to their tight closure. Due to the short time of using the developed dosimeters for measuring UV radiation, the harmful release of microplastics into the marine environment is eliminated. Thus, this work does not consider the influence of UV radiation and water salinity on the degradation of polystyrene capsules and foils.

## 4. Conclusions

The work concerns the development of capsule dosimeters for the actual assessment of UV radiation doses (UVA and UVB) in sea and ocean waters in areas of coral reefs. The methods used so far to assess UV radiation doses in sea and ocean waters are based on the use of radiometers and satellite systems for tracking changes in the intensity of high-energy radiation, which register separately for each of the UVA and UVB subranges. The TTC-Pluronic F-127 dosimeters developed in this work are chemical systems that are easy to manufacture, easy to use, do not pose a threat to fauna and flora, and at the same time are easy to read without the need for highly specialised measuring equipment. The developed dosimeters with a concentration of 0.1% TTC can be used in a range of up to 5 J/cm^2^, which predisposes them to UV radiation measurements at a depth of more than 10 m in sea and ocean waters in 10-min intervals during all months throughout the year. Due to the good stability of the dosimeters over time, lasting at least 28 days, the chemical composition can be further refined by adding UV retarders to increase the intervals of use and increase their measurement range. The paper also discusses the method of applying dosimeters under real-world conditions in the form of single capsules or flat packages.

## Figures and Tables

**Table 1:** Photographs of TTC-Pluronic F-127 dosimeters containing different concentrations of TTC unirradiated and irradiated with different doses of UV radiation in the air and seawater.

TTC concentration [% *w*/*w*] (unirradiated samples)	0.1% TTC	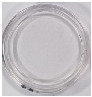	0.05% TTC	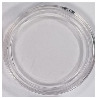
Medium of irradiation	Air	Water	Air	Water
Irradiated with 0.4 J/cm^2^ UV	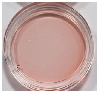	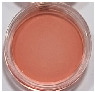	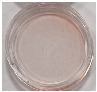	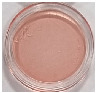
Irradiated with 0.8 J/cm^2^ UV	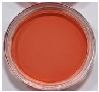	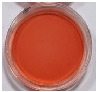	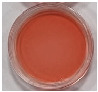	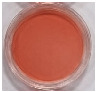
Irradiated with 2 J/cm^2^ UV	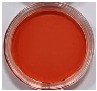	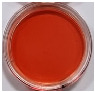	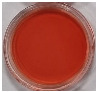	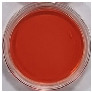

**Table 2:** CIELab parameters for TTC-Pluronic F-127 dosimeters with 0.1% and 0.05% TTC irradiated with UV radiation in air and seawater.

TTC Concentration (% *w*/*w*)		0.1			0.05		
Medium	Dose (J/cm^2^)	L	a	b	L	a	b
air	0	82.09	0.09	3.39	82.82	0.01	3.30
	0.2	80.17	6.01	4.91	81.23	2.89	4.06
	0.4	74.52	15.76	8.36	79.52	5.49	5.15
	0.6	63.32	34.05	16.65	66.44	28.98	14.10
	0.8	57.88	41.56	22.24	59.56	37.85	19.64
	1	55.19	43.58	24.59	56.00	42.36	23.59
	2	53.82	44.70	26.10	51.46	45.22	27.20
	3	51.04	45.94	28.63	51.25	45.78	28.46
	4	49.77	46.51	29.77	49.65	45.86	29.13
	5	48.90	46.67	29.54	47.84	46.56	29.97
water	0	82.17	0.08	3.35	81.61	0.02	3.21
	0.2	76.23	11.90	6.91	80.66	3.75	4.45
	0.4	64.35	30.92	15.23	70.83	21.34	10.67
	0.6	58.45	40.28	21.75	63.60	33.00	16.56
	0.8	55.92	42.51	24.14	58.82	39.42	20.96
	1	53.27	44.89	27.35	56.98	41.79	23.22
	2	51.98	45.23	28.11	51.84	45.57	27.82
	3	50.00	46.21	29.45	50.80	45.68	28.30
	4	48.38	46.02	29.29	50.65	45.58	28.65
	5	47.79	46.34	29.69	49.37	45.68	28.82

**Table 3:** Basic characteristics of TTC-Pluronic F-127 dosimeters irradiated with UV radiation in air and seawater medium derived from the dose responses (Figure 2). The linear equation is for the linear dose range, whereas the exponential equation is for the dynamic dose range of samples irradiated with UV radiation.

TTC (% *w*/*w*)	Medium	Threshold Dose (J/cm^2^)	Dynamic Dose Range (J/cm^2^)	Linear Dose Range (J/cm^2^)	Linear Equation R = a × D + R_0_, Where D Is Dose, R Is Reflectance and R_0_ Is Intercept			Exponential Equation R = R_0_ + Ae^−D/t^, Where A and t Are Constants			
					a Dose Sensitivity (% × cm^2^/J)	R_0_ Intercept (%)	R^2^	R_0_	A	t	R^2^
0.1	air	<0.2	<0.2–4	0–0.6	−82.047 ± 9.290	64.658 ± 4.405	0.963	5.866 ± 0.690	62.228 ± 8.128	0.349 ± 0.053	0.902
	water	<0.2	<0.2–5	0–0.4	−112.673 ± 5.312	61.190 ± 1.991	0.996	5.973 ± 0.182	57.615 ± 4.709	0.236 ± 0.015	0.963
0.05	air	<0.2	<0.2–2	0–0.6	−80.777 ± 23.504	69.880 ± 11.739	0.783	5.366 ± 0.789	66.312 ± 12.046	0.394 ± 0.075	0.836
	water	<0.2	<0.2–2	0–0.6	−81.378 ± 12.209	63.001 ± 5.589	0.935	5.904 ± 0.570	61.490 ± 7.024	0.317 ± 0.034	0.918

**Table 4:** Stability over time of non-irradiated and irradiated dosimeters containing 0.1% TTC stored in air and seawater.

Time after Irradiation	Immediately after Preparation/Irradiation	14 days after Preparation/Irradiation	
		Storage in Water	Storage in Air
Non-irradiated	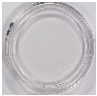	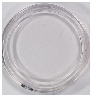	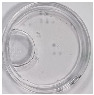
Irradiated with 2 J/cm^2^ UV	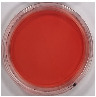	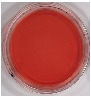	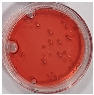

**Table 5:** Mean exposure of UV radiation received at different depths (from 1 to 20 m) at the Red Sea in April, July, September, and December 2017 (according to data from 2).

Month	Dose	Depth (m)		
		1	10	20
April	(J/cm^2^/day)	1102.8	387.2	124.0
	(J/cm^2^/h)	46.0	16.1	5.2
	(J/cm^2^/10 min)	7.7	2.7	0.9
July	(J/cm^2^/day)	1380.8	535.0	191.0
	(J/cm^2^/h)	57.5	22.3	8.0
	(J/cm^2^/10 min]	9.6	3.7	1.3
September	(J/cm^2^/day)	1182.4	406.4	126.0
	(J/cm^2^/h)	49.3	16.9	5.3
	(J/cm^2^/10 min)	8.2	2.8	0.9
December	(J/cm^2^/day)	737.7	203.5	52.0
	(J/cm^2^/h)	30.7	8.5	2.2
	(J/cm^2^/10 min)	5.1	1.4	0.4

## Data Availability

Data are available on request by contacting the corresponding authors.
